# Antibody dynamics and spontaneous viral clearance in patients with acute hepatitis C infection in Rio de Janeiro, Brazil

**DOI:** 10.1186/1471-2334-11-15

**Published:** 2011-01-12

**Authors:** Alexander M Strasak, Arthur Y Kim, Georg M Lauer, Paulo S de Sousa, Cleber F Ginuino, Carlos A Fernandes, Carlos E Velloso, Adilson J de Almeida, Jaqueline M de Oliveira, Clara F Yoshida, Julian Schulze zur Wiesch, Gláucia Paranhos-Baccalá, Stefan Lang, Larry J Brant, Hanno Ulmer, Susanne Strohmaier, Lalit Kaltenbach, Elisabeth Lampe, Lia L Lewis-Ximenez

**Affiliations:** 1 Department of Medical Statistics, Informatics and Health Economics, Innsbruck Medical University, Innsbruck, Austria; 2 Division of Infectious Diseases, Massachusetts General Hospital and Harvard Medical School, Boston, USA; 3 Gastrointestinal Unit, Massachusetts General Hospital and Harvard Medical School, Boston, USA; 4 Viral Hepatitis Laboratory, Oswaldo Cruz Institute, FIOCRUZ, Rio de Janeiro, Brazil; 5 Hepatitis Division, Central Public Health Laboratory Noel Nutels, Rio de Janeiro, Brazil; 6 Hematology Unit, Gaffrée & Guinle University Hospital, Federal University of the State of Rio de Janeiro, Rio de Janeiro, Brazil; 7 Viral Technology Laboratory, Oswaldo Cruz Institute, FIOCRUZ, Rio de Janeiro, Brazil; 8 Medizinische Klinik I, Universitätsklinikum Eppendorf, Hamburg, Germany; 9 Emerging Pathogens Laboratory, Fondation Merieux, Lyon, France; 10 Institute of Statistics, University of Innsbruck, Innsbruck, Austria; 11 Gerontology Research Center, National Institute on Aging, Baltimore, USA

## Abstract

**Background:**

The anti-HCV antibody response has not been well characterized during the early phase of HCV infection and little is known about its relationship to the clinical course during this period.

**Methods:**

We analyzed serial anti-HCV antibodies longitudinally obtained from a prospective cohort of 65 patients with acute HCV infection by using a microparticle enzyme immunoassay AxSYM HCV 3.0 (Abbott Diagnostics) during the first 12 months from HCV acquisition in Rio de Janeiro, Brazil. Spontaneous viral clearance (SVC) was defined as undetectable HCV RNA in serum, in the absence of treatment, for three consecutive HCV PCR tests within 12-months of follow-up.

**Results:**

Baseline antibody values were similar among patient groups with self-limiting HCV evolution (n = 34) and persistent viremia (n = 31) [median (interquartile range) signal/cut-off ratio (s/co) 78.7 (60.7-93.8) vs. 93.9 (67.8-111.9), p = 0.26]. During 12-months follow-up, patients with acute spontaneous resolving HCV infection showed significantly lower serial antibody response in comparison to individuals progressing to chronic infection [median (interquartile range) s/co 62.7 (35.2-85.0) vs. 98.4 (70.4-127.4), p < 0.0001]. In addition, patients with self-limiting HCV evolution exhibited an expeditious, sharp decline of serial antibody values after SVC in comparison to those measured before SVC [median (interquartile range) s/co 56.0 (25.4-79.3) vs. 79.4 (66.3-103.0), p < 0.0001].

**Conclusion:**

Our findings indicate a rapid short-term decline of antibody values in patients with acute spontaneous resolving HCV infection.

## Background

Acute hepatitis C virus (HCV) infection accounts for approximately 20% of cases of acute hepatitis today, with an estimated 30,000 to 40,000 new cases occurring every year in the United States alone. Worldwide at least 170 million individuals are chronically infected with HCV [1-4]. The natural history of HCV infection is heterogeneous and incorporates a range of prognostic determinants [1-6]. Untreated, acute HCV infection progresses to chronic infection in 50-80% of patients [7-9]. Rates of spontaneous HCV resolution (SVC) reported from prospective studies substantially vary, with estimates ranging from 10 to 60% [2-6,10,11].

As acute HCV infection is clinically inapparent in most cases, longitudinal data on the natural course of early disease remain sparse [12] with the immunologic correlates of spontaneous recovery being poorly understood [13]. While antibodies detected by commercially available tests, have been widely used for diagnosing HCV infection, there is little information on the timing, magnitude, specificity and clinical relevance of the antibody dynamics during acute HCV infection, and its relation to short-term disease outcome widely remains unclear [1,2,14-19]. We here present detailed 12-month follow-up data on serial antibody values in a Brazilian cohort of 65 patients with acute HCV infection, followed prospectively from the initial phase, between 2001 and 2009. We compared longitudinal patterns of antibody ratios between individuals with self-limiting acute HCV evolution and patients progressing to chronic infection.

## Methods

We have recently published the results of an acute HCV cohort in Rio de Janeiro, Brazil [20], which showed an independent relationship of peak anti-HCV antibody levels and disease outcome. However, in this study we extended the study prospectively to 12 months and used serial anti-HCV antibody ratios obtained from a commercial microparticle enzyme immunoassay (MEIA) AxSYM HCV 3.0 (Abbott Laboratories) and serial qualitative HCV RNA detected by the Cobas Amplicor Monitor HCV test (Roche Diagnostics) for analysis. Patients who did not clear HCV RNA during early follow-up were referred for antiviral therapy. Six of the patients in the present study underwent antiviral treatment within the first 12 months from infection and their anti-HCV antibody ratios were not considered for analysis during and after the treatment period. A more detailed description of the program methodology and study cohort has been reported previously [20].

This study was approved by the Research on Human Subjects Ethics Committee of the Oswaldo Cruz Foundation as well as the Brazilian National Research Ethics Commission. Signed informed consent was obtained from all participants.

### Laboratory Methods

Following diagnosis of acute HCV infection, samples were obtained from study participants at approximately every two weeks for the first, second and third month and then monthly from the fourth month to one year between 2001 to 2009. Overall 85% of scheduled blood draws were obtained. Serum samples obtained serially were aliquoted for serological and molecular testing and stored at -80°C. Samples were thawed only once for laboratory testing. Repeat tests were performed for anti-HCV antibody testing or HCV RNA detection on separate samples obtained at the same time point. Anti-HCV antibody testing results were obtained from ratios between sample absorbance and the calculated cut-off for each sample (s/co) with the automated MEIA AXSYM HCV 3.0. The qualitative determination of HCV RNA was carried out by the Cobas Amplicor Monitor HCV test (Roche Diagnostics) which has a detection limit of 50 IU/ml. First time samples that were HCV RNA undetectable by the Cobas Amplicor Monitor were retrospectively reevaluated by the VERSANT HCV RNA Qualitative Assay (TMA) (Siemens Healthcare Diagnostics) with a lower detection limit of 9.6 IU/ml.

### Definitions

Diagnosis of acute HCV infection was based upon previously established criteria [10,11,21]: (1) a positive anti-HCV antibody or HCV RNA result in a patient with a negative anti-HCV test result within the past year, or (2) a positive anti-HCV and HCV RNA result in a patient with clinical hepatitis, ALT levels 10 times the upper limit of normal (32 U/L); or, (3) in absence of detectable HCV RNA, history of high-risk exposure between 1 and 3 months prior to clinical manifestation in anti-HCV seropositive patients. Further details on the diagnosis of acute HCV infection is described elsewhere [20]. To estimate the date of HCV infection the day of high-risk exposure was used, or, when unavailable, as either 6 weeks before the onset of symptoms in symptomatic patients [7,10], or 6 weeks before seroconversion in asymptomatic patients [10,15].

Spontaneous viral clearance (SVC) was defined as undetectable HCV RNA in serum within the first 12 months of follow-up after the estimated date of infection, in the absence of treatment. Since oscillations in HCV RNA detection are frequently observed in the early phase of HCV infection, two additional consecutive negative HCV RNA test results were required to sustain SVC classification [22,23]. The midpoint between the date of the first of three consecutive RNA-negative samples and the date of the last positive HCV RNA was used as the estimated date of SVC. For six patients with undetectable HCV RNA in the first sample collected, the date of SVC was estimated as the midpoint between the date of infection and the date of the first undetectable HCV RNA sample [10,11].

### Statistical Analysis

Inter-individual differences in baseline antibody values (i.e. measurements at first visit) and median antibody values during prospective 12-months follow-up between patients with self-limiting and chronic HCV evolution were assessed using independent samples t-test. Serial patterns of longitudinal antibody values between patient groups were analyzed by means of linear mixed-effects regression models, utilizing intra-individual differences and multiple measurements per patient over time. Two-sided p-values < 0.05 were considered statistically significant. All analyses were performed using STATA 10.0 and SAS 9.1 statistical software.

## Results

### Patient characteristics

40 women (61.5%) and 25 men (38.5%) comprised the cohort eligible for analysis. Mean age at HCV infection was 45.7 years (range, 20-77 years). 54/65 patients (83.1%) experienced disease symptoms (including jaundice and/or dark urine) during follow-up. During the 12-month follow-up 439 serial antibody measurements were obtained (median per patient 6.0, range 1-16). Antibody values were approximately normally distributed, with a sample absorbance/cut-off ratio (s/co) ranging from 0.4 to 185.6. After 12 months of follow-up, 34/65 patients (52.3%) had spontaneously recovered from acute HCV, including 5 with clearance after six months of follow-up. A detailed comparison of sociodemographic and clinical characteristics between individuals with acute spontaneous resolving HCV infection and patients' progression to chronic infection has been previously reported by our group [20].

### Longitudinal Antibody Response and Spontaneous HCV Clearance

Baseline antibody ratios were similar among patient groups with self-limiting HCV evolution (n = 34) and persistent viremia (n = 31) [median (interquartile range - IQR) s/co ratio 78.7 (60.7-93.8) vs. 93.9 (67.8-111.9), p = 0.26, Table 1]. Compared to patients with persistent viremia, patients with self-limiting HCV evolution had significantly lower median and serial antibody ratios [105.5 s/co (IQR 75.4-123.6) vs. 66.2 s/co (IQR 47.8-79.5), p < 0.0001, and 98.4 s/co (IQR 70.4-127.4) vs. 62.7 s/co (IQR 35.2-85.0), p < 0.0001, Table 1]. In addition, patients with self-limiting HCV infection showed a significant and sharp decline of serial antibody ratios after SVC [79.4 s/co (IQR 66.3-103.0) before SVC vs. 56.0 s/co (IQR 25.4-79.3) after SVC, p < 0.0001, Table 1].

**Table 1:** Anti-HCV antibodies (s/co) during first 12 months of follow-up in 65 patients with acute HCV infection, stratified according to viral clearing status, Rio de Janeiro, Brazil, 2001-2009.^a^

Anti-HCV Antibodies (s/co)	**SVC**^**b **^**(n = 34)**			Non-SVC (n = 31)	p-value SVC vs. Non-SVC	p-value before SVC vs. after SVC
			
	Total	Before SVC	After SVC			
At Baseline/First Visit	78.7 (60.7-93.8)	n.a.^c^	n.a.	93.9 (67.8-111.9)	0.26	n.a.
Median during follow-up	66.2 (47.8-79.5)	71.0 (55.8-80.0)	55.1 (17.9-71.8)	105.5 (75.4-123.6)	<0.0001	0.04
Serial during follow-up^d^	62.7 (35.2-85.0)	79.4 (66.3-103.0)	56.0 (25.4-79.3)	98.4 (70.4-127.4)	<0.0001	<0.0001

^a ^Data given as median (interquartile range).

^b ^Spontaneous Viral Clearance (SVC) was defined as a series of at least 3 negative HCV RNA results within 12 months after the estimated point of infection.

^c ^n.a. denotes not applicable.

^d ^All anti-HCV antibody measures per patient during the first 12 months from the estimated date of infection were considered and weighted equally.

P-value for group comparison calculated from linear-mixed effects regression.

## Discussion

We present the antibody dynamics during the initial phase of disease in 65 individuals with acute HCV infection, prospectively followed in Rio de Janeiro, Brazil. Although different profiles of long-term humoral immune response between spontaneous clearers and chronic carriers have previously been described in HCV infection [13,17-19,24,25], the dynamics of humoral responses during the acute phase of infection are less well documented due to the fact that acute HCV cohorts and prospective data from the early phase of disease are rare [12]. Our previous study had demonstrated that low levels of anti-HCV are predictive of spontaneous viral clearance in the acute phase of infection in patients followed during a 6-month period. This study, however, in extending the study to a 12-month time period, identified an additional 5 cases of SVC and shows that longitudinal antibody response may be used as a predictor of spontaneous viral during early phase of HCV infection when rapid declines in anti-HCV antibodies occur. From a clinical standpoint, two or more serial values may be more feasible than examining peak antibody values [20] as a discriminator of outcome.

Takaki and colleagues [13] analyzed the HCV-specific humoral immune responses in patients infected with HCV over an 18-20 year course of infection after documented exposure and found that a large number of patients with viral clearance tested negative for anti-HCV antibodies. Messick and colleagues [24] studied a cohort of haemophiliacs exposed to HCV infection and observed significant decrease in anti-HCV antibody ratios in patients with viral clearance when compared to those with persistent viremia over a period of 15 years. Lu and co-workers [25], described persistently low antibody s/co ratios in subjects with spontaneous viral resolution, suggesting that antibody ratios might not rise significantly in those who spontaneously recover.

It has been speculated that the partial or total loss of anti-HCV antibodies in immunocompetent patients that have spontaneously recovered from HCV may be attributed to the lack of HCV antigen that would sustain antibody levels [24,26]. Studies in chimpanzees have already shown that low doses of HCV inoculum may promote cellular immune responses in chimpanzees but rarely produce detectable viremia or seroconversion [27]. Cross-sectional studies have additionally demonstrated that indeterminate and weak antibody reactivity are each important predictors of absence of HCV viremia [28]. Our data provide additional information regarding the timing of emergence of this antibody pattern, showing that the HCV-specific humoral immune response declines early after clearance, once antigenic stimulus is removed.

Our study does have potential limitations that should be considered. First, patients in our cohort were predominantly symptomatic and of female sex, with both characteristics previously being shown to be associated with favourable outcome in acute HCV infection [6]. Second, exact date of exposure was not available for all patients and had to be estimated on the basis of established criteria. However, sensitivity analyses previously conducted by our group [20] indicated that the impact of this limitation is negligible. Finally, sample size eligible for the present analyses was relatively small and time periods between prospective follow-up visits and numbers of serial antibody measures varied among study participants, preventing us from evaluating antibody responses at uniform time points for all patients during follow-up.

## Conclusions

Our data indicate a rapid short-term decline of antibody values in patients with acute spontaneous resolving HCV infection, with the dynamics of this decline being significantly faster than previously appreciated. These findings suggest that HCV antibody testing performed years after infection might be less reliable than currently thought as a marker for HCV exposure, with underestimation of the rate of HCV infection and spontaneous clearance. Serial anti-HCV antibody ratio measurements may also distinguish outcome and could be useful for prognosis in settings where HCV RNA testing is unavailable or constrained by resource limitations.

## List of abbreviations

HCV: hepatitis C virus; SVC: spontaneous viral clearance; ALT: alanine aminotransferase; s/co: sample absorbance to cut-off ratio

## Conflicts of interest

All authors: no conflicts

## Individual Authors 'contributions

AMS, AYK, GML, JSzW, GPB, EL, LLL: Conception and design of study

PSF, CFG, CAF, CEV, AJA, JMO, CFTY, LK, EL, LLL: Acquisition of data and data management

AMS, SL, LJB, HU SS, LK, LLL: Statistical analysis and interpretation of data

AMS, AYK, GML, JSzW, SS, LLL: Drafting of manuscript

AMS, AYK, GML AJA, JSzW, HU, SS, LLL: Critical revising of manuscript for important intellectual content

All authors read and approved the final manuscript.

## Pre-publication history

The pre-publication history for this paper can be accessed here:

http://www.biomedcentral.com/1471-2334/11/15/prepub
